# Functional Activity of the Antioxidant System of *Artemisia* Genus Plants in the Republic of Buryatia (Russia) and Its Significance in Plant Adaptation

**DOI:** 10.3390/plants13182630

**Published:** 2024-09-20

**Authors:** Svetlana V. Zhigzhitzhapova, Elena P. Dylenova, Danaya B. Goncharova, Bato V. Zhigzhitzhapov, Elena A. Emelyanova, Anastasiya V. Polonova, Zhargal A. Tykheev, Selmeg V. Bazarsadueva, Anna S. Taraskina, Evgeniya T. Pintaeva, Vasiliy V. Taraskin

**Affiliations:** 1 Baikal Institute of Nature Management, Siberian Branch of the Russian Academy of Sciences, Ulan-Ude 670047, Russia; zhig2@yandex.ru (S.V.Z.); danaydomi5@gmail.com (D.B.G.); zhbat120401@gmail.com (B.V.Z.); emelianowa.elena2312@mail.ru (E.A.E.); shinigami_n@mail.ru (A.V.P.); gagarin199313@gmail.com (Z.A.T.); bselmeg@gmail.com (S.V.B.); astaraskina@mail.ru (A.S.T.); e-pintaeva@yandex.ru (E.T.P.); vvtaraskin@binm.ru (V.V.T.); 2 Filippov Buryat State Agricultural Academy, Ulan-Ude 670024, Russia

**Keywords:** Baikal region, semiarid conditions, *Artemisia*, superoxide dismutase, catalase, total phenolic content, total flavonoid content, antiradical activity, essential oils

## Abstract

Plants are sessile organisms and any changes in environmental factors activate various responses and defense mechanisms. *Artemisia* plants widely inhabit harsh conditions of arid and semiarid ecosystems. Using two species—a subshrub, *Artemisia frigida*, and an annual–biennial herb, *Artemisia scoparia*—the functioning of the antioxidant system of plants in semiarid territories have been examined. The activity of enzymatic antioxidants and the content of non-enzymatic antioxidants in both species as well as the antiradical activity of their extracts have been shown. Although the plants were collected in areas differing in moisture supply, the activity of enzymatic antioxidants and the content of non-enzymatic antioxidants corresponds to their physiological level, within the range of the norm of reaction, in wormwood. Consequently, conditions of differing moisture deficiency do not cause a specific biochemical response at the level of the antioxidant system in the studied species, which confirms their adaptability to these conditions. Meanwhile, *A. frigida* plants show greater morphological and biochemical plasticity than *A. scoparia* under changing growth conditions. Both species contain tissue monoterpenoids and sesquiterpenoids, the emission of which provides additional protection against high temperatures and drought. Their composition and contents of phenolic components illustrates the differences in adaptation between perennial and annual plants.

## 1. Introduction

One of the key problems of our time is the change in climatic conditions across the planet. Since plants lead a sessile lifestyle, even short-term changes in environmental factors activate various responses and defense mechanisms. Active oxygen species (AOS), by products of aerobic metabolism, are important signaling molecules in the regulation of numerous plant development processes. One of the first responses to stress is the increased production of AOS. Excessively generated reactive oxygen species (ROS) are neutralized by components of the universal, i.e., responsive to heterogeneous stress factors, antioxidant defense system (ADS) [1,2]. An increased activity of antioxidant enzymes in response to stress helps to reduce the degree of damage caused by oxidative stress [3].

In the long-term adaptation of plants, the main “players” ensuring effective protection of metabolism from ROS are low molecular weight antioxidants—ascorbic acid, glutathione, phenolic compounds, proline, soluble carbohydrates, tocopherol, and carotenoids [4]. The role of the ADS lies in maintaining a delicate balance between ROS production and their removal pathways [5,6]. Continuous exposure to a complex of stressors leads to the adaptation of plants to them, with the baseline levels of ADS components, generated under certain environmental conditions, being higher in plants growing in more severe conditions. An important role in the extracellular protection of plants from the damaging effects of oxidants is played by volatile compounds of a terpene nature, particularly mono- and sesquiterpenoids. A certain reserve of these compounds is contained in plant tissues and has long been used under the name essential oils.

Stress conditions in plants can be caused by a number of factors, including lack of water, excess water, and salinity [7,8]. The study of plants in semiarid areas in natural populations will make it possible to show the limits of plant resistance to drought. Stress triggers a response in the morphological and biochemical reactions of plants [9,10]. Today, drought is one of the main stress factors and plants are developing various adaptive mechanisms and strategies to increase their tolerance to water shortage [10]. To understand these mechanisms, it is useful to study plants growing under natural conditions, as shown in the following works [11,12].

The uniqueness of this territory of Buryatia is associated with the presence of the largest freshwater reservoir, Lake Baikal, as well as its geographical location in the center of Asia and its elevation above sea level. Buryatia belongs to the eastern sector of the most continental part of the semiarid zone of Central Asia. This zone marks the western boundary of the Asian anticyclone’s stationary area. The second longitudinal boundary, separating it from the Pacific region, is also defined by the stationary area of the Asian anticyclone. As the Pacific monsoons move from east to west, the amount of moisture they bring decreases, but the timing of precipitation remains stable, with more than 75% of the annual total occurring in the second half of summer [13]. As a result, plants are water-stressed for most of the growing season.

The Baikal region is part of the Angarsk floristic center of *Artemisia* origin and constitutes one of the loci of the greatest diversity of *Artemisia* plants in Eurasia. The genus complex of wormwood in Buryatia is represented by 48 species [14]. Wormwoods play a significant role in steppe ecosystems and are the first to colonize disturbed lands, forming almost pure thickets. Plants of this genus contain a wide range of antioxidant compounds—phenolic compounds, essential oils, ascorbic acid, and others. Due to the rich chemical composition of wormwood, substances derived from it have wide prospects for creating products in the pharmaceutical, food, agricultural, and cosmetic industries [15,16,17]. The species diversity of wormwoods and their ecological plasticity make them convenient models for identifying correlations in “components of ADS—climate”.

Plant species with different life forms and, accordingly, different life strategies will have different levels of activity of ADS components. In this paper we have studied the characteristics of ADS functioning of two species of wormwood in the natural and climatic conditions of Buryatia

## 2. Results

### 2.1. Morphological Observations

The morphometric parameters of *Artemisia frigida* plants from the Cheremukhovaya narrow valley area were similar to those from the Khorinsky and Eravninsky districts of Buryatia, with the number of generative shoots ranging from nine to 16 (Table 1).

In contrast, the plants from the Ivolginsky district, in the foothills of the Ganzurinsky ridge, and the Selenginsky district were smaller and had only one to three generative shoots. This can be explained by the fact that plants from the Khorinsky and Eravninsky districts grow in more moisture-rich eastern regions, while plants from the Cheremukhovaya narrow valley area in 2023 were located near groundwater outflows. The stunted habitus of *A. frigida* growing in the Selenginsky district, in addition to the lower moisture availability of their habitats, may be related to grazing by cattle. On the other hand, *A. scoparia* from different habitats showed almost no external differences, even plants collected in rock crevices (AS-3), although plants from the Khorinsky district were smaller in habitus due to narrower inflorescences.

Overall, the habitus of *A. frigida* was more variable compared to *A. scoparia.*

### 2.2. Lipid Peroxidation Level

Lipid peroxidation (LPO) is an indicative reaction of cell membrane damage [18]. Unsaturated fatty acids are the most vulnerable to ROS, particularly free radicals. The LPO level of fatty acids in lipids results in the formation of peroxide compounds and aldehydes, including the toxic malondialdehyde (MDA), which reacts with thiobarbituric acid (TBA-reactive substances, TBA-RS). LPO occurs when free radicals attack the hydrogen atoms of the methylene group in the alkyl chain, which is conjugated with two double bonds of unsaturated fatty acids. The resulting fatty acid peroxide radical involves a new fatty acid molecule in the process [19]. LPO processes threaten cell membranes because they can completely lose their barrier function as a result.

**Figure 1:**
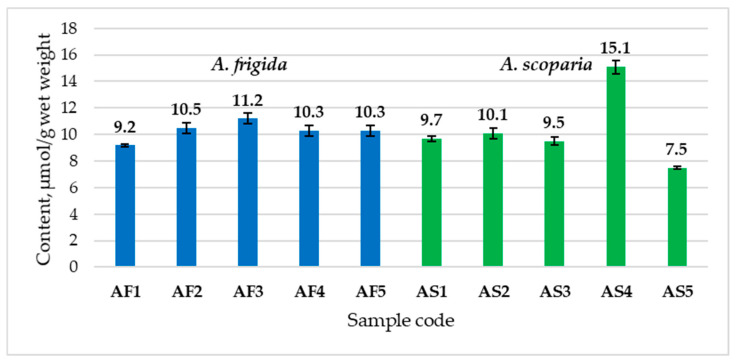
Content of TBA-reactive products in the aerial parts of *A. frigida* and *A. scoparia*, in µmol/g wet weight (MEAN ± SD).

The lowest and highest TBA-RS content was noted in samples of *A. scoparia* from the eastern regions of the Republic: from the Eravninsky district (7.5 μmol/g of raw mass, AS5) and from the Khorinsky district (15.1 μmol/g of raw mass, AS4). The TBA-RS content in samples of *A. frigida* ranged from 9.2 to 11.2 μmol/g of raw material. The lowest TBA-RS content was noted in plants of *A. frigida* growing in the Cheremukhovaya narrow valley (9.2 μmol/g of raw mass, AF1), and the highest in those from Selenginsky district (11.2 μmol/g of raw mass, AF3). At the same time, some increase in TBA-RS in *A. frigida* samples from Selenginsky district (AF3) correlated with a more depressed habitus of the plants compared to other samples (Figure 1).

### 2.3. Antioxidant Plant Enzymes

One of the most important antioxidant enzymes are superoxide dismutase (SOD) and catalase (CAT). SOD is an antioxidant enzyme that provides the first line of defense against ROS within the cell. It is responsible for catalyzing the dismutation of the superoxide anion free radical by converting it into hydrogen peroxide and molecular oxygen. The primary pool of hydrogen peroxide in the cell is broken down by CAT, which decomposes hydrogen peroxide into water and molecular oxygen. CAT is highly specific to hydrogen peroxide and has low activity towards organic peroxides [20].

The activity of the indicated enzymes in both species of wormwood from the flora of Buryatia was significantly different (*p* < 0.05; Table 2). In *A. frigida*, the highest SOD activity was observed in plants from the Cheremukhovaya narrow valley area (7.4 U/mg protein, AF1), while the lowest activity was found in plants from the foothills of the Ganzurinsky ridge (0.2 U/mg protein, AF2). In *A. scoparia*, SOD activity ranged from 0.1 to 0.4 U/mg protein. The highest CAT activity in *A. scoparia* was observed in plants from the foothills of the Ganzurinsky ridge (7.9 μmol H_2_O_2_/mg protein·min, AS2), and the lowest was in plants from the surroundings of the Oninoborsk village (4.3 μmol H_2_O_2_/mg protein·min, AS4). In *A. frigida*, the highest CAT activity was found in plants collected in the surroundings of the Novoselenginsk village (3.8 μmol H_2_O_2_/mg protein·min, AF3), and the lowest in plants from the Cheremukhovaya narrow valley (0.7 μmol H_2_O_2_/mg protein·min, AF1). Overall, *A. frigida* plants exhibited higher SOD activity compared to *A. scoparia* plants from the same habitats. Conversely, CAT activity was higher in *A. scoparia* plants compared to *A. frigida* plants. No correlation between SOD and CAT activities was found in *A. frigida*, although there was a high correlation (r = 0.8) between these enzymes in *A. scoparia*.

### 2.4. Non-Enzymatic Plant Antioxidants

#### 2.4.1. Ascorbic Acid and Glutathione Content

In the long-term adaptation of plants, low molecular weight antioxidants play a crucial role in efficiently protecting metabolism from ROS and other free radicals. These primarily include ascorbic acid and glutathione [4].

Ascorbic acid interacts with free radical forms of ROS, singlet oxygen, and hydrogen peroxide. The antioxidant effect of glutathione arises from its direct interactions with ROS and exchange reactions with proteins containing disulfide bonds, along with the functioning of various enzymes in the glutathione cycle. Glutathione can stabilize membrane structures by removing acyl peroxides formed during lipid peroxidation [18].

Studies on the content of ascorbic acid and the accumulation of reduced glutathione in the aerial parts of *A. frigida* and *A. scoparia* showed that both of these antioxidants’ levels differ between the two species. Specifically, the content of ascorbic acid and reduced glutathione in the aerial parts of *A. frigida* are higher than those in the aerial parts of *A. scoparia* by approximately 1.5 and 1.3 times, respectively (Table 3).

#### 2.4.2. Total Phenolic Content and Total Flavonoid Content

**Figure 2:**
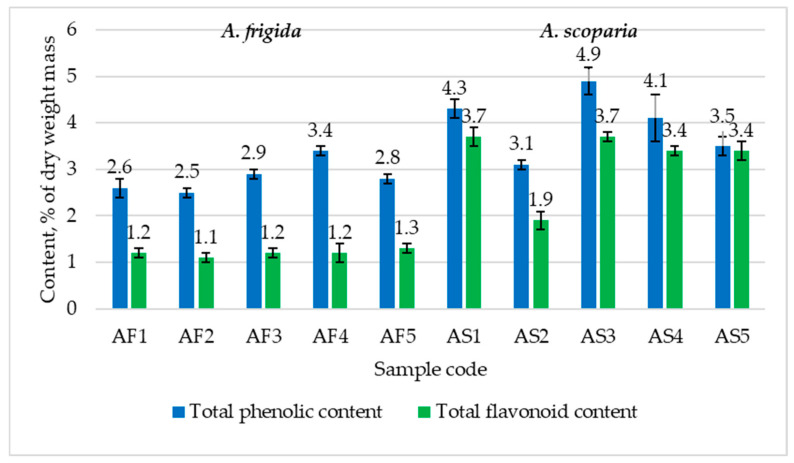
TPC and TFC in *A. frigida* and *A. scoparia* of Buryatian flora (collected in 2023), % of dry weight mass (MEAN ± SD).

The lowest total phenolic content (TPC) in *A. frigida* was observed in sample AF2 (Ivolginsky district, foothills of the Ganzurin Ridge), while the highest was in AF4 (Khorinsky district, surroundings of the Oninoborsk village). The total flavonoid content (TFC) in *A. frigida* samples was more uniform and ranged from 1.1 to 1.3%. The highest TPC and TFC in wormwood was found in sample AS3 (Selenginsky district, surroundings of the Novoselenginsk village), while the lowest was in AS2 (Ivolginsky district, foothills of the Ganzurin Ridge). Overall, the total content of phenolic compounds (both TPC and TFC) was higher in *A. scoparia* samples (*p* < 0.05; Figure 2).

### 2.5. Antiradical Activity of Alcohol Extracts

**Figure 3:**
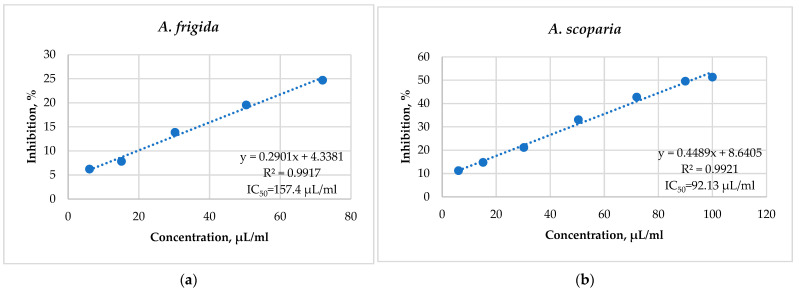
DPPH Antiradical activity of alcohol extracts of *A. frigida* (**a**) and *A. scoparia* (**b**).

The IC_50_ values of alcohol extracts of *A. frigida* and *A. scoparia* were 157.4 µg/mL and 92.13 µg/mL, respectively. Alcohol extracts of *A. scoparia* exhibited greater antioxidant (antiradical) activity (Figure 3).

### 2.6. Composition of Essential Oils by GC-MS and PCA-Analysis

**Figure 4:**
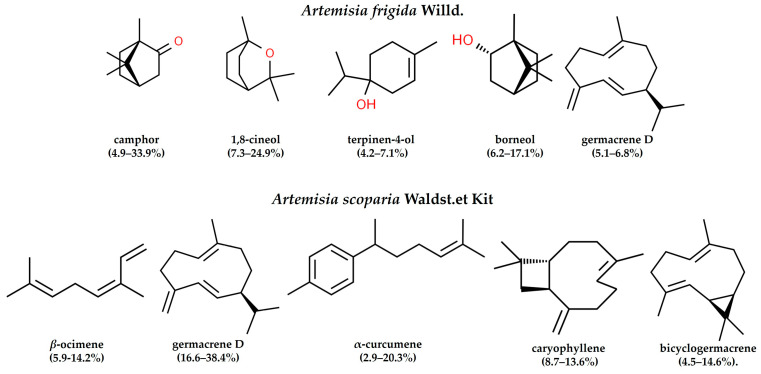
Major components of the EO of *A. frigida* and *A. scoparia* from the Buryatian flora.

According to the analysis made by the GC-MS method, the composition of essential oils (EO) of *A. frigida* from Buryatian flora, collected in 2023, comprises 30–92 compounds, with 13 components being constantly present, identified in all samples regardless of the plant’s habitat. Dominant components include monoterpenoids such as camphor, 1,8-cineol, terpinen-4-ol, borneol, and sesquiterpene germacrene D. The composition of the EO of *A. scoparia* from the flora of Buryatia consists of 22–31 mono- and sesquiterpenoids, with 18 components being constantly present. Dominant components include the monoterpenoid *β*-ocimene and sesquiterpenoids germacrene D, α-curcumene, caryophyllene, and bicyclogermacrene (Figure 4).

**Figure 5:**
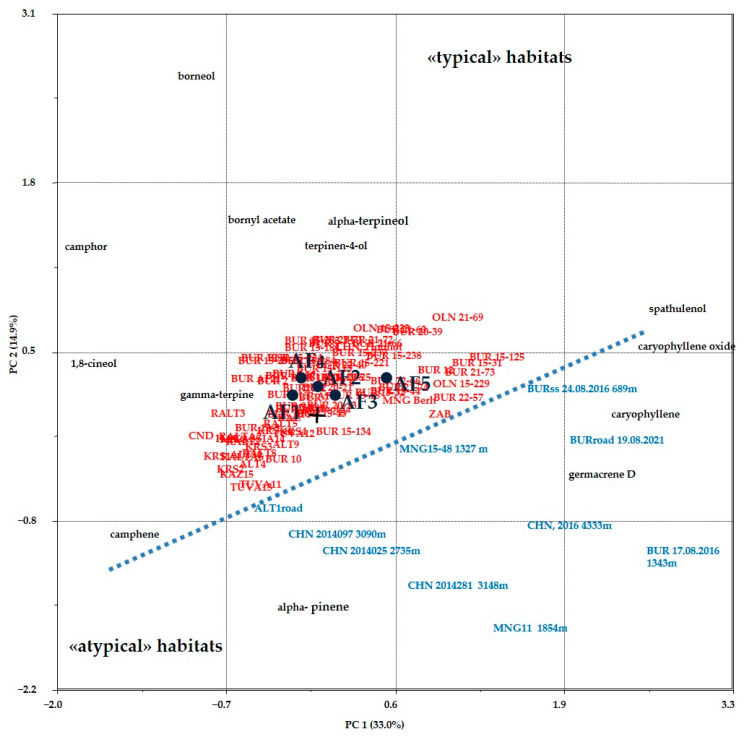
Biplot (PC1–PC2) of data on the content of constant compounds in the EO of *A. frigida* plants from different regions of the world (Principal Component Analysis). “AF1-5”—data published for the first time in this article, other designations—previously published data [21]. The biplot shows the compounds whose content in EO influences the division of plant samples into groups of “typical” and “atypical” habitats.

In our previous study [21], it was found that the EO of *A. frigida* plants growing in “typical” habitats for *A. frigida*, i.e., on light and rocky soils with relatively sparse vegetation, differ in the content of dominant components from the EO of plants from “atypical” habitats [22]. “Atypical” habitats of *A. frigida* are associated with altitudes above 1300 m above sea level (Figure 5, samples from Mongolia are designated MNG, from Buryatia—BUR, from Qinghai—CHN with the designation of the altitude above sea level at which the raw materials were collected) and the proximity of roads (BURroad and ALTroad) [23]. In the conditions of Transbaikalia, on the southern sunlit slopes, more water evaporates, and plant communities experience a moisture deficit [24,25], leading to differences in the composition of EO (sample BURss). In the present work, we have supplemented the data on the content of constituents, essential oils of *A. frigida* from the article [21], with the data included in the present study and have shown, using the principal components method, that the composition of essential oils corresponds to that characteristic of plants from “typical” habitats (Figure 5).

Based on our previous analysis of own and literature data, we proposed to classify the composition of the main components of the EO of *A. scoparia* into the following chemotypes: (A) “acetylene hydrocarbons” (plants from the northern provinces of Iran, Tajikistan, and the European part of Russia); (B) “monoterpenoid and aromatic compounds” (plants from the eastern provinces of Iran, India, and South Korea); and (C) “monoterpenoid or aromatic and sesquiterpenoid compounds” (Kazakhstan, Mongolia, Buryatia) [26]. The results obtained in 2023 show that the components of *A. scoparia* are represented by mono- and sesquiterpenoid compounds and correspond to the third chemotype, characteristic of semiarid and arid territories.

## 3. Discussions

We have noted a greater variability in the habitus of *A. frigida* plants compared to *A. scoparia*. Other authors have also observed the high plasticity of *A. frigida* plants, which, depending on the conditions of their habitat, form various morphological types ranging from low-growing densely pubescent forms to relatively tall semi-shrubs with long annual vegetative and generative shoots [22,27,28,29].

However, the adaptation of plants in arid ecosystems occurs not only at the morphological and physiological levels but also at the biochemical level. These mechanisms are based on maintaining of a delicate balance between ROS production and their removal pathways. ROS is present in plants under normal (physiological) conditions [6]. The functional interaction of different types of antioxidants, in our opinion, helps to maintain a spatial–temporal balance between the generation and inactivation of ROS (ROS homeostasis), ultimately ensuring the adaptation of plants to their growing conditions. Otherwise, a stronger ROS signal would induce programmed cell death [4]. At the same time, changes in the intensity of ROS formation activate reactions associated with plant morphogenesis, and reactions of hypersensitivity and apoptosis are controlled by ROS. Finally, ROS influence the activity of protein molecules through sensory proteins [30].

A vivid example of ROS action on cell membranes is LPO. A certain level of LPO is constantly present even under normal cell conditions [31,32]. Our estimated values of TBA-RS are significantly higher than the content of MDA in *A. annua* and close to that in *Tanacetum vulgare* and *Matricaria chamomilla*, growing in Uzbekistan [33]. At the same time, our results are close in values to the intensity of LPO in cucumber and radish seedlings under the influence of heavy metals [34] or in the leaves of winter wheat plants growing under the simultaneous influence of low temperatures and optimal or high concentrations of zinc [35]. All of the above indicates that while the results of TBA-RS content cannot provide an exact quantitative characteristic of stress, they unequivocally indicate the presence of oxidative stress in the model populations. Moreover, they reflect the natural level of action of environmental stress factors, since prolonged exposure to sublethal levels of stress factors can lead to adaptive changes in plant cells, as is the case in our study.

Thus, if our reasoning is correct, long-term protective mechanisms should be developed in wormwood plants growing in the territory of Buryatia, and the antioxidant system of plants should have higher indices of functional activity, with a higher constitutive pool of antioxidants compared to plants in humid territories.

Therefore, the first line of defense against ROS inside the cell is the enzyme SOD [36]. Any change in the water regime of plants, either towards drought or overhydration, is stressful for plants and triggers the organism’s adaptive protective responses. However, it is not always in response to stress that the activity of SOD changes in a unidirectional manner; it depends on both the intensity and duration of the stress exposure and the plant’s stress tolerance [37,38,39]. Therefore, the high activity of SOD in the AF1 sample may be explained by a sharp change in the local conditions of their habitat, as the *A. frigida* plants in this population found themselves in a place where groundwater emerged to the surface this year. Perhaps the sudden increase in soil moisture caused a xerophytic imbalance in *A. frigida* plants between ROS content and antioxidants and activated SOD, aimed at immediate protection against ROS inside the cell. In mesoxerophytic species of *A. scoparia* in the same conditions, there was no noticeable increase in SOD activity. *A. scoparia*, being an annual or biennial weed, in our opinion, is less susceptible to changes in soil moisture, and therefore we do not observe a significant change in SOD activity in the specified species in the Cheremukhovaya narrow valley (AS1).

As a result of SOD functioning, hydrogen peroxide is formed, which is an oxidant. Therefore, the next line of defense is an enzyme with high specificity to hydrogen peroxide—CAT. Its main function is located at peroxisomes, where it catalyzes the two-step dismutation of hydrogen peroxide, forming water and molecular oxygen. At the same time, the existence of CAT isoenzymes indicates its diverse role in plant cells. There is also a concept that CAT proteins play a role in removing metabolic hydrogen peroxide in peroxisomes, and the accumulating array of genetic data has demonstrated the important role of CAT in immune reactions and development reactions associated with oxidative signaling. Some pathogens target CAT activity to alter ROS metabolism and thus modulate or suppress host immunity [40,41,42]. All of the above explains the absence of a linear correlation between the activities of SOD and CAT enzymes in perennial semi-shrub polycarpic plants of *A. frigida*, unlike in annual herbaceous monocarpic plants of *A. scoparia*.

On the other hand, the increase in intracellular hydrogen peroxide levels may have a greater impact on the total pools of ascorbate ion and tissue glutathione and is a consequence of enhanced participation of catalase-independent pathways [43]. The high content of both non-enzymatic antioxidants ascorbic acid and glutathione, in our opinion, compensates for the low activity of CAT against the background of high SOD activity in *A. frigida* plants growing in the Cheremukhovaya narrow valley, since both investigated antioxidants contribute to the decomposition of hydrogen peroxide. They also remove the peroxyl radicals of fatty acids formed during lipid peroxidation, to which CAT has a lower affinity. Thus, *A. frigida* plants from the Cheremukhovaya narrow valley area demonstrate a biochemical stress response to sudden changes in soil moisture, manifested by an increase in SOD activity. Both investigated species of Buryatian wormwood are comparable in terms of ascorbic acid content with *Glaucium flavum*, and in terms of reduced glutathione content with *Euphorbia peplus* [44].

In conditions of constantly acting or periodically repeating combinations of excess temperature and light, as well as stress induced by drought, isoprenoids and phenolic compounds can also complement the functional roles of antioxidant enzymes [45]. The close values of TPC and TFC in the samples of *A. scoparia* can be explained by the fact that the Folin–Ciocalteau reagent reacts with many reducing substances. Flavanols and flavonones demonstrate the highest reducing capacity in reactions with this reagent [46]. In contrast, aluminum chloride selectively reacts only with flavonoids that have hydroxyl groups in position 3 and/or 5 [47].

Indeed, the main flavonoids for species of *Artemisia* from the *Frigidae* subsection (*Absinthium* section), including *A. frigida*, are flavones of various structural types, such as apigenin, luteolin, chrysoeriol, and tricin [48,49]. On the other hand, for *A. scoparia* (Scopariae section), flavonols such as kaempferol, quercetin, rutin, hyperoside, and others are predominant [50,51,52]. Therefore, in the case of *A. scoparia*, the TPC is close to the TFC (Figure 2).

In addition to primary actions the antioxidant activity of phenolic compounds can also be explained by their ability to chelate trace elements, inhibiting enzymes involved in the formation of ROS [53]. Higher contents of TPC and TFC in *A. scoparia* plants may also explain the lack of significant differences in the activities of enzymatic antioxidants in *A. scoparia* plants collected in different parts of the Republic of Buryatia.

Several authors suggest that phenolic compounds are the main carriers of antioxidant activity in alcohol extracts [11,54], among which polyphenolic compounds are the most active antioxidants [1]. On the one hand, since the highest TPC was observed in *A. scoparia* plants (Figure 2), their alcohol extracts show the highest antioxidant activity compared to those of *A. frigida*. It is known that flavonols are more active in the presence of the diphenylpicrylhydrazyl radical than flavones [55]. This also explains the higher antiradical activity of alcohol extracts of *A. scoparia* compared to those of *A. frigida* (Figure 3).

To test our hypothesis of higher levels of functional activity and antioxidant content in plants of semiarid systems, we conducted an analysis of literary data. In this regard, we encountered the following difficulties. We did not find data on the activity of enzymes in wormwood, and we can only say that the SOD activity of Buryatia wormwood is comparable to that of cotton plants (1.2–2.7 U/mg protein) [38] and birch leaves (1.2–8.1 U/mg protein) [20]. The CAT activity of the studied species is comparable to the activity of catalase in chickpea (2 μmol H_2_O_2_/mg protein), *Arabidopsis* under hypothermia (2.5 μmol H_2_O_2_/mg protein), saffron (15 μmol H_2_O_2_/mg protein), and cabbage (18 μmol H_2_O_2_/mg protein) [56].

We can only state that the TFC in the aboveground part of *A. frigida* is higher than in the leaves and inflorescences of the same species in Western Siberia (Novosibirsk region, Omsk region). The TFC in *A. scoparia* plants from natural habitats of Buryatia is higher compared to plants from Novosibirsk region and close to the values in plants from the Omsk region and Altai Republic [57].

Volatile terpene compounds act as the first line of extracellular defense under conditions of high air temperatures and drought. These compounds form a cloud around the plant, while scavenging ROS, thereby protecting plant cell membranes from LPO [58]. We have shown that there is a “core” of EO components, the biosynthesis of which for a particular species is genetically predetermined [21]. The composition of the “core” of *A. frigida* includes monoterpenoids in large quantities, in particular camphor, 1,8-cineole, terpineol-4, and borneol. It is also known that both damaged and undamaged *A. frigida* plants release them into the atmosphere [59,60]. Specifically, the emission of monoterpenes protects plants from severe drought periods, providing an additional barrier against plant damage during acute water shortages [61].

Conversely, the main components of the EO of *A. scoparia* are sesquiterpenoids. Sesquiterpenes are known to more effectively neutralize ozone than monoterpenes. For example, sesquiterpene (E)-β-caryophyllene is 43 times more reactive compared to the monoterpene limonene. Sesquiterpenes (E)-β-farnesene and (E)-α-bergamotene have a high potential for protection against abiotic stress [62]. The protective mechanism is associated both with direct reactions of terpenoids with oxidants, either intracellularly or at the boundary between the leaf and the atmosphere/boundary layer, stabilizing the membrane, and with indirect changes in ROS signal transduction [63]. At the same time the release of sesquiterpenes from leaves is one of the mechanisms by which plants cope with oxidative burst, one of the manifestations of the adverse effects of these abiotic factors, meaning their emission increases at the onset of water deficit conditions, under relatively mild water stress conditions [61].

Our results on the composition of EO and the TFC and TPC align with the lifespan of both species—a perennial subshrub and herbaceous annual, since *A. frigida* needs to adapt annually to recurring adverse factors to maintain viability, while *A. scoparia* only needs to “survive” the adverse period once in its life.

## 4. Materials and Methods

### 4.1. Description of Studied Area

The territory of the Republic of Buryatia features a highly complex morphostructure, characterized by alternating mid-altitude mountains with wide intermontane basins of predominantly tectonic or erosion-accumulative origin [64].

Plant collection was carried out in the southern, central, and eastern parts of Buryatia, which slightly differ in their main climatic parameters. To characterize the main climatic parameters, specialized data provided by the All-Russian Research Institute of Hydrometeorological Information—World Data Center (RIHMI-WDC) were used: air temperature and precipitation during the growing season (May–August) over five years (2019–2022), and their integral characteristics based on data from three meteorological stations (Table 4). Additionally, integral characteristics were calculated: the Selyaninov hydrothermal coefficient (HTC), the temperature–moisture extremity coefficient (K_ext_), the Palmer Drought Severity Index (PDSI), and the Standardized Precipitation–Evapotranspiration Index (SPEI).

To characterize the level of heat and moisture supply of the territories, HTC was calculated using the formula: HTC = R × 10/Σt, where R represents the total precipitation in millimeters with an average daily temperature above +10 °C, and Σt denotes the sum of the average daily temperatures in degrees Celsius (°C) for the same period. To compare the growing conditions of plants, the extremity coefficient K_ext_ was calculated, representing the ratio of the average temperature value over a specific period to the amount of precipitation for the same period (K_ext_ = t °C/mm). The PDSI is based on the “supply–demand” concept of the water balance equation, accounting for precipitation deficits and location specifics. The SPEI takes into account both precipitation and evapotranspiration when assessing moisture conditions (Table 4).

The Eastern districts of Buryatia differ from the Central and Southern districts in terms of the amount of precipitation and temperatures, as well as the average temperature during the growing season. The Eastern districts of Buryatia experience higher moisture availability due to greater precipitation during the growing season and moderate temperatures. The integral index values (HTC, SPEI, PDSI) also indicate better moisture conditions in the Eastern districts of Buryatia during the growing season. The extremity coefficient confirms the greater extremity of plant growing conditions due to high temperatures in the summer months in the Southern and Central districts of Buryatia.

Overall, the territory of Buryatia does not experience severe or extreme droughts, and it is in the zone of mild droughts. However, over five years, there have been significant fluctuations in the amount of precipitation, and changes in integral characteristic values have been observed, ranging from mild drought to excessive moisture, reflecting the changing modern climate. The Transbaikal region, including the steppe areas of Buryatia, is in a wet period characterized by an increased level of atmospheric precipitation. However, there is no temporal stability in the long-term climate changes overall, and it is forecasted that due to the cyclical nature of precipitation and increasing air temperatures, moisture deficiency in Transbaikalia will lead to an increase in potential forest fire danger and a decrease in agricultural crop yields [65].

### 4.2. Plant Materials

Plant material for analysis was collected from the natural habitats of the plants in the Republic of Buryatia in August 2023, during the flowering phase of both *Artemisia* species (Table 5).

One portion of the plant material was dried according to the standard procedures for drying plant raw materials [66] and used to determine non-enzymatic antioxidants. Another portion of the plant material was immediately frozen in liquid nitrogen at the collection site and stored frozen until the analysis of enzymatic antioxidant activity and the measurement of lipid peroxidation levels.

*Artemisia frigida* Willd. is a subshrub which acts as a dominant or co-dominant species in the steppes of Siberia, the Volga region, Kazakhstan, Central Asia, Mongolia, and North America. *Artemisia scoparia* Waldst. et Kit. is a herbaceous plant that usually grows on fallows, fields, open slopes, and borders [67]. Although the plants grew in neighboring populations, *A. frigida* was collected from steppe areas, while *A. scoparia* was collected from areas adjacent to paths in open spaces in the surroundings of the Novoselenginsk village, on the southern slope of a mountain, in rock crevices, and between stones.

Morphometric measurements were conducted on 50 randomly selected plants. The following morphometric parameters were measured for *A. frigida*: plant height, base diameter of the shrub, and number of generative shoots. For *A. scoparia*, plant height, and the width and length of inflorescences were measured.

### 4.3. Measurement of Lipid Peroxidation

The Thiobarbituric Acid Reactive Substances (TBA-RS) assay is a well-established method for monitoring lipid peroxidation levels, and is measured as described in [18].

Frozen samples (0.5 g) were taken and ground in 5 mL of phosphate buffer (pH 7.4). Then, 0.3 mL of extraction solution, 1 mL Thiobarbituric Acid (TBA), 3 mL of orthophosphoric acid, and 0.1 mL of FeSO_4_ solution were mixed. The mixture was heated at 95–100 °C in a constant temperature water bath for 30 min and then cooled in cold water (10 °C) to room temperature. Afterwards, 4 mL of n-butanol was added to each sample, shaken, and centrifuged at 10,000 rpm for 10 min. The supernatant was detected at 532 (maximum absorbance of TBA-RS and 600 nm (correction for nonspecific turbidity). When determining the concentration of TBA-RS (µmol mL^−1^ fresh weight), we used extinction coefficient (ε), equal to 155 mM^−1^ s^−1^.

### 4.4. Enzymic Antioxidants

#### Measurement of Superoxide Dismutase (SOD, EC 1.15.1.1.) and Catalase (CAT. EC 1.11.1.6) Activities

Enzyme extracts for SOD and CAT activities were prepared by freezing the weighed amount (0.5 g) of samples in liquid nitrogen to prevent proteolytic activity, followed by grinding with 1.5–2.0 mL extraction buffer (0.1 M phosphate buffer, pH 7.8). The enzyme extract was centrifuged for 20 min at 15,000 rpm at 4 °C, and the supernatant was collected and assayed for enzyme activity.

The total SOD activity was determined according to method 1 described by [18]. To a 3 mL reaction mixture containing 0.1 M phosphate buffer (pH 7.8), 39 μM methionine, 0.245 μM nitro blue tetrazolium, 0.3 μM EDTA, 0.1% Triton X-10, and 0.1 mL of supernatant was added 0.5 mL riboflavin. The tubes were shaken and illuminated and allowed to run for 10 min after which the light was switched off and the absorbance read at 560 nm. One unit of SOD activity was defined as the amount of enzyme required to cause 50% inhibition of the rate of nitroblue tetrazolium chloride reduction. Activity was expressed as units/min/g protein (U/min/g protein).

The CAT activity was measured according to the method described by [18]. The CAT assay mixture of 3 mL consisted of 0.2 mL supernatant, 0.1 M phosphate buffer (pH 6.8–7.0), 33% H_2_O_2_. A decrease in the absorbance was recorded at 240 nm. The CAT activity was expressed as μmol of H_2_O_2_ oxidized per minute per gram protein.

All the samples were analyzed in triplicate.

### 4.5. Non-Enzymic Antioxidants

#### 4.5.1. Determination of Ascorbic Acid and Glutathione

The ascorbic acid and glutathione contents were determined by titrimetry according to [18]. A ground, powdered plant sample (300 mg) was extracted with 5% trichloroacetic acid. An aliquot (5 mL) of the extract was added to test tube. Titrimetric determination of ascorbic acid was carried out with 2,6-dichlorophenolindophenol, glutathione with potassium iodate.

#### 4.5.2. Determination of Total Phenolic Content, Total Flavonoid Content

The dried and ground powdered sample (1 g) was extracted with 100 mL of 70% aqueous ethanol (*v*/*v*), heated in a water bath for 60 min at reflux. The hot extract was filtered through a ashless filter paper into a 100 mL volumetric flask.

The TPC in the extract was spectrophotometrically determined by the Folin–Ciocalteau assay, using gallic acid as a standard [68]. The reaction mixture was prepared by mixing 1 mL of extract and adjusted to 10 mL of distilled water. 5 mL of Folin–Ciocalteau reagent was added to 1 mL of extract and shaken, kept in the dark for 5 min, then, 4 mL of 7.5% sodium carbonate was added and incubated at room temperature for 60 min in the dark. In parallel, a blank reagent was prepared using distilled water. Absorbance was measured relative to the prepared reagent blank at 760 nm on a multimodal microplate reader CLARIOstar Plus (BMG LABTECH GmbH, Ortenberg, Germany). The concentration of the TPC in the extract was expressed as a % of the weight of the air-dry raw material (% by weight of the air-dry substance).

The TFC was determined using aluminum chloride assay according to [69]. Briefly, an aliquot (1 mL) of the extract was added to a 25 mL test tube. To each test tube, 2 mL of 2% alcohol solution AlCl_3_ and one drop of 30% acetic acid, 96% ethanol (to the mark) were added. Preliminarily measured absorption spectra of the aluminum chloride complex with alcoholic extracts from the aerial parts of *A. frigida* plants showed the presence of a main maximum in the range of 397 nm, which corresponds to a complex with luteolin, *A. scoparia* in the range of 410 nm, which corresponds to the rutin complex. Therefore, the absorbance of the resulting solution was measured at 397 nm (*A. frigida*), at 410 nm (*A. scoparia*). Luteolin (*A. frigida*), rutin (*A. scoparia*) was used as standard to express the TFC of samples. The concentration of TPC in the extract was expressed as a % by weight of the air-dried raw materials.

#### 4.5.3. Composition of Essential Oils

EOs were obtained by hydrodistillation from air-dry raw materials (above-ground part of plants, for 3 h) and its component composition was determined by gas chromatography–mass spectrometry as described in [70].

### 4.6. Antiradical Activity

The antiradical activity of alcohol extracts was determined by the DPPH test (using a stable radical, 2,2-diphenyl-1-picrylhydrazyl). Briefly, a DPPH solution (0.006% in 95% ethanol) was added to alcohol extracts and incubated for 30 min in the dark at room temperature. The antiradical activity was then determined spectrophotometrically on a CLARIOstar Plus (BMG LABTECH GmbH, Ortenberg, Germany) multimodal microplate reader at 517 nm.

The antiradical activity (in % inhibition) was calculated using the formula:

% inhibition of DPPH radicals = [(A_0_ − A_1_)/A_0_] × 100,

where A_0_ is the absorbance of the control sample, and A_1_ is the absorbance of the test sample.

The IC_50_ index was determined using regression analysis.

### 4.7. Statistical Analysis

All samples were randomly selected. The data collected were processed and averaged across sampling groups using MS Excel 2016 (Microsoft Corporation, Redmond, WA, USA). The mean and SD were calculated for all sampling groups per species. The Shapiro-Wilk test was used to test the normal distribution of the samples. The paired Student’s *t*-test was used to compare the means of two normally distributed samples. The Wilcoxon signed-rank test was used to compare the means of other samples. Calculations were performed using built-in functions of the R programming language (version 4.2.1).

The built-in functions of MS Excel 2016 (Microsoft Corporation, Redmond, WA, USA) were used to study the antiradical activity of ethanol extracts.

The Principal Component Analysis (PCA) method was also used to analyze the content of essential oil constituents using Sirius software version 6.0 from Pattern Recognition Systems, a/s, Norway [71]. Relative values (i.e., percentage of the sum) of EOs components were logarithmically transformed. It is allowed us to derive an equation that could be used to define quantitative differences among individual compounds. As a scaling method, the unit or unit variance scaling was applied, which is common and uses standard deviation as the scaling factor [72].

## 5. Conclusions

Using two species of *Artemisia* with different life forms and, consequently, different life strategies—the xeropetrophytic steppe subshrub *A. frigida* and the mesophytic herbaceous weedy annual *A. scoparia*, we have examined the peculiarities of the functioning of the antioxidant system of plants in semiarid and arid territories.

The presence of oxidative stress in plants within the model populations indicates the presence of lipid peroxidation products. Inside the plant cells, enzymatic and non-enzymatic components of the ADS protect the plants from the damaging effects of excessive AOS production. Since the components of ADS of *A. scoparia* displayed little correlation with habitat conditions, it is a sign that the parameters are still within the range of the norm of reaction.

At the same time, *A. frigida* plants demonstrate greater morphological and biochemical plasticity compared to *A. scoparia* when their growing conditions change. For instance, *A. frigida* plants that encountered an area with groundwater emergence this year have a more developed habitus and greater SOD activity, as well as higher levels of ascorbic acid and glutathione. The levels of TPC and TFC in both wormwood species remain more constant. However, *A. scoparia* plants are characterized by higher TPC and TFC levels and greater activity of alcoholic extracts. Overall, the TFC content in both species is higher than in plants growing in other subhumid regions. Both studied wormwood species contain tissue monoterpenoids and sesquiterpenoids, whose emission provides additional extracellular protection against high temperatures and drought.

The healthy appearance of the plants of both species shows that they are adapting to the natural conditions of Buryatia. The characteristics of components of the ADS of *A. frigida* are also within the reaction norm. Consequently, habitat conditions varying in degree of moisture deficiency do not cause a specific biochemical response at the level of the ADS in the studied species, which confirms their adaptability to semiarid conditions of Buryatia.

The composition of EOs and contents of TPC, TFC illustrates the differences in adaptation between perennial and annual plants to semiarid climate. Thus, annual *A. scoparia* accumulates sesquiterpenoids and more phenolic compounds in their tissues, while perennial *A. frigida* plants, adapted to cyclically recurring adverse factors, predominantly accumulate monoterpenoids less TPC and TFC.

Our results indicate the important role of the ADS in plant adaptation to environmental conditions. However, more detailed studies are needed, involving observations during ontogenetic development (at least in the latent and active periods), as well as during seasonal development (with the manifestation of four patterns of composition changes in different phenophases) and studies on the qualitative composition of phenolic compounds in Buryatia’s wormwood species.

## Figures and Tables

**Table 1:** Morphometric parameters of the studied *Artemisia* Species, flowering phase (2023).

Sample Code	Morphological Parameters		
	Plant Height, mm *	*A. frigida*—Diameter of the Bush Base, cm; *A. scoparia*—Inflorescence Length, mm.	*A. frigida*—Number of Generative Shoots; *A. scoparia*—Inflorescence Width, mm.
*A. frigida*			
AF1	246.8 ± 27.1	128.5 ± 19.1	12.2 ± 3.0
AF2	211.1 ± 35.4	117.4 ± 28.3	2.6 ± 1.4
AF3	212.9 ± 36.3	123.5 ± 30.3	1.9 ± 1.3
AF4	247.7 ± 30.2	162.3 ± 20.9	13.3 ± 2.7
AF5	268.7 ± 18.3	160.4 ± 19.4	13.8 ± 2.8
*A. scoparia*			
AS1	390.8 ± 81.4	240.2 ± 30.8	135.9 ± 26.1
AS2	385.7 ± 77.5	230.0 ± 35.1	145.3 ± 22.1
AS3	388.8 ± 80.6	236.2 ± 22.8	136.9 ± 22.8
AS4	386.5 ± 79.1	236.2 ± 29.0	122.1 ± 26.5
AS5	391.7 ± 81.5	239.0 ± 28.0	135.1 ± 25.8

* MEAN ± SD.

**Table 2:** Superoxide dismutase (SOD, U/mg protein) and catalase (CAT, μmol H_2_O_2_/mg protein·min) activities in the fresh mass of aerial parts of *A. frigida* and *A. scoparia* from the Buryatian flora (2023).

Sample Code	*A. frigida*					*A. scoparia*				
	AF1	AF2	AF3	AF4	AF5	AS1	AS2	AS3	AS4	AS5
SOD	7.40 ± 0.51 *	0.2 ± 0.01	0.7 ± 0.03	0.8 ± 0.02	1.4 ± 0.04	0.4 ± 0.02	0.4 ± 0.02	0.1 ± 0.01	0.2 ± 0.01	0.2 ± 0.02
CAT	0.7 ± 0.03	1.0 ± 0.04	3.8 ± 0.03	3.2 ± 0.03	1.8 ± 0.04	6.5 ± 0.04	7.9 ± 0.05	4.8 ± 0.03	4.3 ± 0.03	6.2 ± 0.04

* MEAN ± SD.

**Table 3:** Content of ascorbic acid (μg/g of fresh mass) and reduced glutathione (mMol/g of fresh mass) in the aerial parts of *A. frigida* and *A. scoparia* from the Buryatian flora (Collected in 2023, Ivolginsky district, Cheremukhovaya narrow valley).

Plant Species	Ascorbic Acid, μg/g of Fresh Mass	Glutathione, mMol/g of Fresh Mass
*A. frigida*	64.3 ± 0.6 *	64.5 ± 0.5
*A. scoparia*	42.9 ± 0.2	50.2 ± 0.9

* MEAN ± SD.

**Table 4:** Climatic parameters and their integral characteristics during May–August 2019–2022 in the Central (Meteorological Station 30823 Ulan-Ude), Southern (Meteorological Station 30825 Ivolginsk), and Eastern (Meteorological Station 30745 Sosnovo-Ozersk) districts of Buryatia.

Meteorological Station (Index, Name)	Climatic Parameters	Year			
		2019	2020	2021	2022
30823 Ulan-Ude	∑t (°C)	1794.9	1731	1655.3	1641.5
	∑ precipitation (mm)	195.3	164	219.7	123.8
	HTC	1.08	0.95	1.33	0.75
	PSEI	0	−0.38	1.42	−0.28
	PDSI	1.46	1.34	1.69	−1.92
	T_av. daily_ (°C)	19.5	18.8	18.0	17.8
	K_ext_ (°C/mm)	0.1	0.11	0.08	0.14
30825 Ivolginsk	∑t (°C)	1742.6	1675	1603.6	1620.2
	∑ precipitation (mm)	134.7	144.9	234.1	100.8
	HTC	0.77	0.86	1.46	0.62
	PSEI	−0.16	0.38	1.87	−0.49
	PDSI	−0.84	−0.91	3.95	−2.38
	T_av. daily_ (°C)	18.9	18.2	17.4	17.6
	K_ext_ (°C/mm)	0.14	0.13	0.07	0.17
30745 Sosnovo-Ozersk	∑t (°C)	1466	1405	1443.7	1405.3
	∑ precipitation (mm)	146.3	275.5	155.8	154.9
	HTC	0.10	1.96	1.08	1.10
	PSEI	−0.34	0.23	0.92	−0.32
	PDSI	0.57	−0.29	1.71	−2.00
	T_av. daily_ (°C)	15.9	15.3	15.7	15.3
	K_ext_ (°C/mm)	0.11	0.06	0.10	0.10

**Table 5:** Characteristics of *Artemisia* sample collection sites in 2023 (flowering phase).

Location	Sample	Sample Code	Collection Date	Latitude Longitude
Ivolginsky district, Cheremukhovaya narrow valley *	*A. frigida*	AF1	23 August 2023	51°93′ N 106°48′ E
	*A. scoparia*	AS1		
Ivolginsky district, foothill of Ganzurinsky ridge	*A. frigida*	AF2	24 August 2023	51°42′ N 107°12′ E
	*A. scoparia*	AS2		
Selenginsky district, surroundings of the Novoselenginsk village **	*A. frigida*	AF3	24 August 2023	51°06′ N 106°39′ E
	*A. scoparia*	AS3		
Khorinsky district, surroundings of the Oninoborsk village	*A. frigida*	AF4	30 August 2023	52°24′ N 110°00′ E
	*A. scoparia*	AS4		
Eravninsky district, surroundings of Mozhayka village	*A. frigida*	AF5	30 August 2023	52°40′ N 110°78′ E
	*A. scoparia*	AS5		

* populations were located near groundwater outflows; ** the population is used as pasture.

## Data Availability

All data generated or analyzed during this study are included in this published article.

## Abbreviations

ADS	antioxidant defense system
AOS	active oxygen species
CAT	catalase
EO	essential oils
HTC	Selyaninov hydrothermal coefficient
K_ext_	temperature–moisture extremity coefficient
LPO	lipid peroxidation
MDA	malondialdehyde
MEAN	arithmetic mean
PDSI	Palmer Drought Severity Index
RIHMI-WDC	All-Russian Research Institute of Hydrometeorological Information—World Data Center
ROS	reactive oxygen species
SD	standard deviation
SOD	superoxide dismutase
SPEI	Standardized Precipitation–Evapotranspiration Index
TBA-RS	thiobarbituric acid reactive substances
TFC	total flavonoid content
TPC	total phenolic content
